# Comparison Between 3D Heads-Up Display System and Standard Operating Microscope in Diabetic Vitrectomy for Tractional Retinal Detachment

**DOI:** 10.22336/rjo.2025.83

**Published:** 2025

**Authors:** Krinjeela Bazgain, Uday Pratap Singh Parmar, Deeksha Katoch, Basavaraj Tigari, Mohit Dogra, Ramandeep Singh

**Affiliations:** 1 Department of Ophthalmology, Advanced Eye Centre, Postgraduate Institute of Medical Education and Research (PGIMER), Chandigarh, India

**Keywords:** diabetic retinopathy, tractional retinal detachment, 3D heads up display, pars plana vitrectomy, 3D-HUD, BCVA = Best-Corrected Visual Acuity, C3F8 = Perfluoropropane Gas, IJO = Indian Journal of Ophthalmology, IOP = Intraocular Pressure, LogMAR = Logarithm of the Minimum Angle of Resolution, NVI = Neovascularisation of the Iris, OCT = Optical Coherence Tomography, PDR = Proliferative Diabetic Retinopathy, POD = Postoperative Day, PPV = Pars Plana Vitrectomy, PRP = Pan-Retinal Photocoagulation, SF6 = Sulfur Hexafluoride Gas, SOM = Standard Operating Microscope, SPSS = Statistical Package for the Social Sciences, TRD (Tractional Retinal Detachment), USG (Ultrasonography), VEGF (Vascular Endothelial Growth Factor), VH (Vitreous Haemorrhage), 3D HUD (Three-Dimensional Heads-Up Display)

## Abstract

**Background:**

Proliferative diabetic retinopathy (PDR) remains a significant cause of vision loss worldwide, often requiring pars plana vitrectomy (PPV) for tractional retinal detachment (TRD). The introduction of the 3D Heads-Up Display (HUD) system offers ergonomic and visualization advantages.

**Methods:**

This prospective interventional study involved eyes that underwent PPV for TRD. Group A underwent surgery using 3D HUD, while Group B utilised a standard operating microscope (SOM). Outcomes were assessed for surgical time, anatomical success, visual recovery, intraoperative variables, and postoperative complications.

**Results:**

The study included 94 eyes from 90 patients (47 eyes per group). Retinal reattachment was achieved in 100% of cases in both groups at 3 months postoperatively. Mean surgical time was significantly shorter in the 3D HUD group (51.50 ± 24.06 minutes) compared to SOM (67.33 ± 31.27 minutes; p = 0.004). Both groups significantly improved visual acuity, with the 3D HUD showing a superior mean LogMAR BCVA improvement at 3 months. Endoillumination levels were markedly lower with the 3D HUD, reducing the risk of phototoxicity. Surgeons reported greater depth perception, manoeuvrability, and teaching utility with the 3D HUD.

**Discussion:**

In this study, 3D HUD provided anatomical outcomes comparable to SOM while offering shorter surgical times, lower illumination requirements, and favourable visual recovery. These advantages likely stem from enhanced depth perception and improved ergonomics inherent to the 3D system. Overall, our findings support 3D HUD as a safe and efficient alternative for managing complex TRD cases.

**Conclusions:**

The 3D HUD system demonstrated comparable anatomical success, shorter surgical times, enhanced ergonomic benefits, and favourable visual outcomes compared to SOM.

## Introduction

Proliferative diabetic retinopathy (PDR), a severe complication of diabetes, remains a leading cause of vision loss worldwide. Affecting approximately 17 million people, PDR progresses without treatment to advanced stages that often require surgical intervention [**1,2**]. In these advanced stages, vision-threatening complications like vitreous haemorrhage and tractional retinal detachment (TRD) develop, putting patients at high risk of severe vision loss [**3**].

For PDR, pan-retinal photocoagulation (PRP) is widely used to reduce neovascularization by targeting ischemic areas of the retina [**3**]. However, when PDR advances to TRD, surgery is essential. Pars plana vitrectomy (PPV) is the standard procedure in these cases, aimed at removing vitreous haemorrhage (VH) and relieving traction to preserve retinal structure and function [**4**]. Innovations in vitrectomy, including microincisional techniques and higher-speed cutting instruments, have improved surgical outcomes in recent years [**4,5**]. However, PPV remains a challenging procedure that requires precision, particularly as the physical demands of traditional microscope-based surgery can contribute to surgeon fatigue over time.

The introduction of 3D heads-up display (3D HUD) systems is an exciting advancement in vitreoretinal surgery, replacing traditional eyepieces with a high-definition display on a large monitor [**6**]. The 3D HUD provides surgeons with enhanced depth perception, the ability to use lower light levels (reducing retinal phototoxicity), and improved ergonomics [**6**]. This system allows surgeons to maintain a heads-up posture, potentially reducing the musculoskeletal strain associated with lengthy procedures [**7**]. Additionally, it presents a unique advantage in teaching environments, giving surgical teams and trainees access to real-time, detailed views of complex surgical manoeuvres [**7**].

Studies comparing 3D HUD with standard operating microscopes (SOM) in surgeries for conditions like macular holes and rhegmatogenous retinal detachment suggest comparable anatomical and visual outcomes [**8-10**]. For instance, Rani and colleagues found that 3D HUD allowed lower illumination levels while achieving similar success rates in retinal detachment repairs, reducing the risk of retinal damage during surgery [**8**]. Despite these promising results, research specifically comparing 3D HUD to SOM in the context of PDR-related TRD cases is limited, particularly concerning surgical time, anatomical outcomes, and postoperative visual recovery.

This study aims to address these gaps by comparing the surgical and functional outcomes of 3D HUD and SOM in patients with PDR and TRD.

## Methods

This prospective, non-randomized interventional study was conducted at a tertiary care institute. The study was conducted from January 2020 to March 2021 and included 100 eyes from 96 patients with diabetes who met the inclusion criteria. Patients were divided into two groups: Group A (50 eyes) underwent vitrectomy using the 3D HUD system, and Group B (50 eyes) underwent vitrectomy using SOM.

### Study Design

The study design was interventional and non-randomized, reflecting the practical constraints of the operating room environment and surgeon expertise. Group A patients were operated on using the NGENUITY 3D Visualization System by Alcon (TX, USA) [**11**], while Group B patients were operated on using the standard SOM. All surgeries in the 3D HUD group were performed by a single experienced vitreoretinal surgeon (RS). In contrast, surgeries in the SOM group were performed by two experienced vitreoretinal surgeons (DK and MD).

### Inclusion Criteria

Patients included in the study had diabetes with fovea-threatening or involving TRD. They could have vitreous haemorrhage or sub-hyaloid bleed, but patients with vitreous haemorrhage alone, those who had not received pre-operative anti-vascular endothelial growth factor (VEGF) injections, or those unable to follow up were excluded. Of the initially enrolled 100 eyes, six were excluded due to loss to follow-up, leaving a final sample of 94 eyes (47 in each group).

### Preoperative work-up

All patients underwent a comprehensive ophthalmic examination, including best-corrected visual acuity (BCVA) using the Snellen chart, applanation tonometry, slit-lamp examination of the anterior and posterior segments, and indirect ophthalmoscopy. Fundus photography and optical coherence tomography (OCT) were performed to document macular traction and retinal detachment. In non-clear media cases due to VH, ultrasonography (USG) was performed to document the TRD. Data were collected preoperatively and on days 1, 7, 30, and 90 postoperatively. Visual acuity data were converted to the logarithm of the minimum angle of resolution (LogMAR) for statistical analysis.

### Surgical technique

Standard 25G pars plana vitrectomy was performed in both groups. The surgical steps included core vitrectomy, identification, and removal of the posterior hyaloid, and segmentation or delamination of fibrovascular membranes. In cases with retinal breaks, fluid-air exchange with subretinal fluid aspiration was performed, followed by endo laser pan-retinal photocoagulation (PRP) as needed. Internal tamponade with air, gas (C3F8 or SF6), or silicone oil was used based on the extent of retinal detachment. All surgeries were performed under local anaesthesia.

### Intraoperative data collection

Data collected during surgery included the duration of surgery, membrane dissection time, number of iatrogenic retinal breaks, number of intraoperative bleeders, endoillumination percentage, and tamponade agent used. Additionally, a questionnaire was used to evaluate the surgeon’s comfort, depth perception, image quality, and teaching experience.

### Postoperative follow-up

Postoperative evaluations were conducted on day 1, day 7, 1 month, and 3 months. BCVA, intraocular pressure (IOP), and retinal attachment status were assessed during each follow-up visit. Fundus photography, OCT, and USG (if needed) were repeated to evaluate anatomical outcomes. Bleeding on POD1 was reported as loose bleeds, and bleeding noted after POD1 was reported as rebleeds.

### Statistical analysis

The data were analyzed using SPSS version 26 (IBM Inc., Chicago, IL, USA). Continuous variables were compared using the Student’s t-test for normally distributed data and the Mann-Whitney U test for non-normally distributed data. A p-value < 0.05 was considered statistically significant. The sample size was calculated using the Clincalc calculator, with a power of 80% and an error probability of 0.05. However, the sample was reduced to 50 per group due to the COVID-19 pandemic, as approved by the ethics committee.

To account for potential bias introduced by different surgeons performing each technique, we applied a mixed-effects model (hierarchical/multilevel model) in our statistical analysis. This approach enabled us to treat the surgeon variable as a random effect, helping control for variability across surgeons and isolate the effects attributable to surgical techniques. By using this model, we aimed to reduce surgeon-related differences in surgical time, tissue manipulation, and comfort, thereby allowing a more accurate comparison of the outcomes between the 3D HUD and SOM systems.

## Results

### Demographics and clinical characteristics

The study included 94 eyes from 90 patients (66 males, 24 females) with TRD, with a mean age of 49.27 ± 9.51 years. Retinal reattachment was achieved in all 94 eyes (100%) in the 3D HUD and SOM group at 3 months postoperatively (p = 1.0), with no significant difference between the two groups.

### Visual outcomes

The BCVA improved from 1.89 ± 0.488 to 1.14 ± 0.720 post-operatively in the 3D HUD group and from 1.94 ± 0.457 to 1.44 ± 0.622 in the SOM group. These differences in BCVA were statistically significant at both 1 and 3 months postoperatively, as summarised in **Table 1**. On direct comparison, the visual outcome in the 3D HUD group was significantly better than in the SOM group at postoperative days 1, 7, 30, and 90. The findings of the comparative analysis are summarised in **Table 2**.

**Table 1:** Mean value of preoperative and postoperative best corrected visual acuity

	3D HUD	P value	SOM	P value
Preoperative	1.89 ± 0.488		1.94 ± 0.457	
Postoperative day 1	2.09 ± 0.583	0.06	2.29 ± 0.312	0.000
Postoperative day 7	1.68 ± 0.732	0.085	2.02 ± 0.439	0.424
Postoperative day 30	1.23 ± 0.718	0.000	1.58 ± 0.617	0.000
Postoperative day 90	1.14 ± 0.720	0.000	1.44 ± 0.622	0.000

**Table 2:** Mean value of postoperative best corrected visual acuity and its significance

	3D HUD	SOM	P value
Postoperative day 1	2.09 ± 0.583	2.29 ± 0.312	0.031
Postoperative day 7	1.68 ± 0.732	2.01 ± 0.436	0.007
Postoperative day 30	1.23 ± 0.718	1.57 ± 0.614	0.012
Postoperative day 90	1.14 ± 0.720	1.44 ± 0.620	0.034

In the 3D HUD group, 37 eyes (76%) showed visual improvement, of which 30 eyes (63.28%) had visual improvement of two or more lines, compared to 30 eyes (63.82%) in the SOM group showing visual improvement, with 22 eyes (46.80%) demonstrating an improvement of two lines or more. Although the difference was not statistically significant, the 3D HUD group showed a trend toward better visual outcomes.

### Intraoperative findings

The mean surgical time was significantly shorter in the 3D HUD group (51.50 ± 24.06 minutes) than in the SOM group (67.33 ± 31.27 minutes; p = 0.004). Membrane dissection time was also shorter in the 3D HUD group (13.33 ± 13.22 minutes vs. 20.82 ± 17.75 minutes, p = 0.019), indicating more efficient tissue manipulation in the 3D HUD system.

The mean number of intraoperative bleeders was comparable between the two groups (6.52 ± 6.27 in the 3D HUD group vs. 7.12 ± 4.82 in the SOM group, p = 0.593). The mean number of diathermy applications was also similar between the groups (p = 0.590). There was no significant difference in the number of iatrogenic retinal breaks between the groups (p = 0.149), highlighting the safety of both systems. These intraoperative findings are summarised in **Table 3**.

**Table 3:** Intraoperative details and their comparison between the two surgical systems

	3D HUD	SOM	p-value
Mean surgical time (minutes)	51.50 +/- 24.06	67.33 +/- 31.270	0.004
Mean dissection time (minutes)	13.33 +/- 13.22	20.82 +/- 17.747	0.019
Mean number of bleeders	6.52 +/- 6.270	7.12 +/- 4.818	0.593
Mean number of times diathermy was used	5.54 +/- 6.115	5.00 +/- 3.546	0.590
Minimum illumination percentage	37.90 +/- 12.539	96.24 +/- 11.148	0.000
Mean number of iatrogenic breaks	0.20 +/- 0.782	0.42 +/- 0.731	0.149

### Endoillumination

Endoillumination levels were significantly lower in the 3D HUD group (37.90 ± 12.54%) compared to the SOM group (96.24 ± 11.15%, p < 0.001). This reduction in illumination reduced the risk of retinal phototoxicity while maintaining high-quality visualization in the 3D HUD system.

### Postoperative complications

Postoperative complications were comparable between the two groups. Exudative retinal detachment was observed in one patient in the SOM group, which was attributed to extensive intraoperative PRP and resolved within a week. Choroidal detachment was observed in two patients in the SOM groups, likely due to extensive intraoperative pan-retinal photocoagulation. It subsided on its own in four weeks. Raised IOP was noted in 8 eyes (2 in the 3D HUD group and 6 in the SOM group), and low intraocular pressure was reported in one patient in the 3D HUD group on postoperative day one. The cases with high IOP were managed with topical anti-glaucoma medications. The difference in the pre- and postoperative IOP was not statistically significant.

Loose blood was observed in seventeen eyes (10 in the 3D HUD group, seven in the SOM group) on postoperative day (POD), and the patients were managed by keeping them propped up. It did not resolve in any of these eyes, and rebleeds were reported in 20 eyes (12 in the 3D HUS group and 8 in the SOM group) on postoperative day seven. A month after surgery, blood cleared except for five eyes in the 3D HUD group and three in the SOM group. Vitreous lavage and intravitreal anti-VEGF agents were performed in three eyes (two in SOM, one in 3D HUD), while the remaining eyes showed spontaneous resolution by three months. Two more eyes in the 3D HUD group presented with vitreous haemorrhage in the third postoperative month. One of them was managed with vitreous lavage and intravitreal anti-VEGF agents, while the other was given a propped-up position and resolved spontaneously. One patient in the 3D HUS group presented with a post-PPV retinal detachment at POD 30 and also had neovascularisation of the iris (NVI) on POD 90.

No cases of endophthalmitis, neovascular glaucoma, or hyphema were observed. There was no statistically significant difference in postoperative complication rates between the two groups (p = 0.400). The postoperative complications are summarized in **Table 4**.

**Table 4:** Postoperative complications reported at different postoperative intervals

Time period		Exudative Retinal Detachment	Loose blood	Choroidal detachment	Low IOP	High IOP	Post-PPV Retinal Detachment	NVI	Rebleed
Postoperative day 1	3D HUD (eyes)	0	10	0	1	2	-	-	-
	SOM (eyes)	1	7	2	0	6	-	-	-
Postoperative day 7	3D HUD (eyes)	0	-	0	0	1	-	-	12
	SOM (eyes)	1	-	1	0	0	-	-	8
Postoperative day 30	3D HUD (eyes)	0	-	0	0	1	1	-	5
	SOM (eyes)	0	-	0	0	0	0	-	2
Postoperative day 90	3D HUD (eyes)	0	-	-	-	-	1	1	2
	SOM (eyes)	0	-	-	-	-	0	0	0

### Surgeon experience and feedback

In our study, a post-operative questionnaire was administered to operating surgeons to evaluate their experience across various aspects, including comfort, visibility, image quality, depth perception, ease of use, manoeuvrability, and teaching utility. In the 3D HUD group, mean satisfaction scores for these parameters were notably high, averaging 4.74, 4.66, 4.68, 4.73, 4.67, 4.66, and 4.93 out of 5, respectively. Compared with the SOM group, the control group yielded lower scores of 4.60, 4.44, 4.48, 4.37, 4.89, 4.41, and 2.96 in the same areas. Depth perception, manoeuvrability, and teaching scored significantly higher in the 3D HUD group (p < 0.05), while SOM was rated as easier to use. Overall, surgeons expressed greater satisfaction with the 3D HUD system, highlighting its advantages in depth perception, manoeuvrability, and teaching effectiveness, particularly benefiting both seasoned surgeons and trainees. The comparative data from the surgeon’s questionnaire are summarised in **Table 5**.

**Table 5:** Mean values and comparison of the results from the Surgeon’s questionnaire (Total score: 5)

	3D HUD	SOM	P value
Comfort	4.74 +/- 0.381	4.60 +/- 0.571	0.153
Visibility	4.66 +/- 0.618	4.44 +/- 0.787	0.123
Image quality	4.680 +/- 0.331	4.48 +/- 0.735	0.083
Depth perception	4.730 +/- 0.322	4.37 +/- 0.561	**0.000**
Simplicity to use	4.670 +/- 0.399	4.89 +/- 0.308	**0.003**
Maneuverability	4.66 +/- 0.383	4.41 +/- 0.636	**0.019**
Teaching	4.93 +/- 0.303	2.96 +/- 0.450	**0.000**

## Discussion

This study demonstrates comparable anatomical success rates for 3D HUD and SOM in achieving retinal reattachment in diabetic vitrectomy cases with tractional retinal detachment (TRD). The 98% reattachment rate observed in the 3D HUD group and 100% in the SOM group aligns with previously reported outcomes, which document success rates ranging from 67% to nearly 100% in similar surgeries. Rahimy and colleagues [**12**] reported an anatomical success rate of 90.3% with a longer follow-up of 11.2 months in eyes with diabetic TRD​, while Dikopf and colleagues [**13**] documented a 99% success rate in their study of 25G vitrectomy for diabetic TRD​. Additionally, Storey and colleagues’ [**14**] analyses revealed progressively higher reattachment rates with smaller-gauge systems, with 100% success in 27G systems, underscoring the role of evolving surgical tools in enhancing outcomes. The consistency between our findings and these prior studies supports the reliability of both 3D HUD and SOM for reattachment in diabetic TRD, affirming the feasibility of 3D HUD for complex PDR surgeries.

Our study also noted significant postoperative visual improvements across both groups, with slightly higher gains in the 3D HUD group. This trend may be influenced by enhanced visualization and reduced intraoperative illumination with 3D HUD, potentially mitigating retinal phototoxicity [**15**]. Prior research on PDR-related retinal detachment surgeries suggests that careful control of light exposure during surgery can influence visual outcomes by preserving retinal sensitivity​ [**16,17**]. For instance, Rani and colleagues [**8**] demonstrated significant visual gains in both the SOM and 3D HUD groups for rhegmatogenous retinal detachment, showing lower endo-illumination requirements with 3D HUD without compromising anatomical outcomes. Talcott and colleagues [**16**] found that, despite longer peel times for epiretinal membranes with 3D HUD, visual and anatomical outcomes were comparable to those with SOM, suggesting that the extended operative time does not detract from patient outcomes. Together, these findings reinforce the value of 3D HUD for visual recovery in TRD and indicate a potential for reduced phototoxicity due to lower illumination requirements.

In terms of operative time, our results showed that surgeries were notably shorter in the 3D HUD group, particularly during membrane dissection. This aligns with findings from Berquet and colleagues [**18**], who found that a 3D HUD significantly reduced total surgical time in cataract and retinal procedures, attributing this to enhanced visibility and depth perception. Conversely, Romano and colleagues [**15**] reported longer overall surgical times in the 3D HUD group due to frequent adjustments needed for optimal focus and resolution, which may stem from a learning curve​. The discrepancy in operative times across studies likely reflects differences in surgeon experience and procedure types. In our research, the shorter surgical times in the 3D HUD group might be attributed to improved posture, increased illumination, and a wider field of view, which enabled more efficient tissue handling. This efficiency not only benefits the patient by reducing anaesthesia exposure but also improves surgeon ergonomics in the long term, an essential factor given the physical demands of vitreoretinal surgery.

In our study, we did have postoperative complications. Loose blood in the fluid-filled cavity was observed postoperatively in 20 eyes in our study. However, most were resolved, and only three required vitreous lavage. Two eyes in the 3D group presented with vitreous rebleed in the third postoperative month. This might have resulted from either anterior hyaloid vascular proliferation or port site neovascularization. In literature, it has been shown that postoperative vitreous rebleed can occur, and its incidence ranges from 12% to 63% [**19**]. Most of them resolved spontaneously, while 4%- 38% required re-surgery [**19**]. Raised IOP was observed in our patients postoperatively, but they were managed with anti-glaucoma medications, and none of them needed surgical intervention. The complications listed in our study were patient-factor-related and not due to the viewing system.

Rani and colleagues [**8**] developed a “Surgeon’s Satisfaction Score” focusing on image quality, teaching capability, and ergonomic comfort, which also favoured the 3D system. In our study, we assessed the operating surgeon’s comfort by having them complete a postoperative questionnaire after each case, recording their experience with comfort, visibility, image quality, depth perception, ease of use, manoeuvrability, and teaching for each surgical setup. The 3D HUD system was reported to offer enhanced depth perception, manoeuvrability, and effectiveness as a teaching tool, though it was found to be less straightforward to use than the SOM. While comfort, visibility, and image quality were higher with the 3D HUD, these differences were not statistically significant, highlighting the system’s ergonomic benefits and potential for educational use. Berquet and colleagues [**18**] noted issues with eye strain, termed “3D asthenopia”, and discomfort from 3D glasses, indicating potential drawbacks for some surgeons. 3D asthenopia was not reported by the surgeon in our study. Furthermore, Berquet and colleagues [**18**] reported significant image latency during anterior segment surgeries but not during posterior segment surgeries. However, no considerable latency or discomfort was observed in our cohort, potentially due to prior experience with the 3D HUD system among our surgeons.

The enhanced ergonomic design of the 3D HUD significantly improved surgeon comfort. Previous surveys of vitreoretinal surgeons have highlighted high rates of musculoskeletal strain from prolonged use of standard operating microscopes [**20,21**]. Venkatesh and colleagues [**22**] found that 49% of Indian ophthalmologists reported low back pain linked to surgery, and 33% reported neck pain, conditions exacerbated by traditional microscopic posture requirements​. In contrast, the heads-up positioning offered by 3D HUD has been shown to reduce the risk of these musculoskeletal issues. Eckardt and colleagues [**6**] reported decreased eye strain with 3D HUD use, and similar ergonomic benefits were noted by Zhang and colleagues [**23**], who highlighted enhanced comfort and depth perception for surgeons​. The superior comfort scores reported by surgeons in our 3D HUD group suggest that, with broader adoption, 3D HUD may help reduce occupational strain among vitreoretinal surgeons, supporting longer, more effective surgical careers [**24,25**].

### Limitations

The main limitation of our study was the use of different surgeons for each operating system, which may have introduced biases in surgical time, tissue manipulation, and surgeon comfort. Although the mixed-effects model was employed to mitigate this by accounting for surgeon variability, it may not eliminate the impact of individual surgeon preferences or skill levels on outcomes. Consequently, some variability in outcomes may still be influenced by differences in surgeon experience rather than the surgical technique alone. Additionally, variations in TRD distribution, initial visual acuity, ischemic changes, and disease chronicity in PDR cases could affect visual outcome assessments, although these factors could not be controlled. While the study’s sample size was initially calculated to be 70 eyes per group for 80% power, the COVID-19 pandemic reduced patient availability, and the sample size was adjusted to 50 eyes per group with approval from the institutional ethics and thesis committees.

## Conclusion

This study reinforces the clinical viability of 3D HUD as an alternative to SOM in complex PDR surgeries. With similar anatomical outcomes and favourable visual recovery, along with ergonomic and educational benefits, 3D HUD presents a valuable advancement in vitreoretinal surgery. The technology’s potential to reduce surgeon strain, mitigate phototoxicity, and enhance training opportunities positions it as a promising tool in managing advanced diabetic retinopathy. Further large-scale studies across diverse surgical contexts will be essential to establishing 3D HUD as a standard for vitreoretinal procedures, thereby improving outcomes for both patients and surgical teams.
